# Economic and health-related quality of life impacts of posttraumatic stress disorder: a systematic review

**DOI:** 10.1186/s13561-026-00800-5

**Published:** 2026-06-02

**Authors:** Tran Hoang Tien, Tran Thu Ngan, Mai Xuan Thu, Meenakshi Sharma, Grainne Crealey, Ciaran O’Neill

**Affiliations:** 1 School of Pharmacy, University of Maryland, Baltimore, USA; 2 Centre for Public Health, Queen’s University Belfast, Belfast, BT12 6BA UK; 3 Health Strategy and Policy Institute, Hanoi, Vietnam; 4 Belfast, UK

## Abstract

**Background:**

Post-traumatic stress disorder (PTSD) is a severe psychiatric condition associated with long-term mental health impairment, high healthcare utilization, and substantial societal costs. Despite growing recognition of its burden, few reviews integrate economic and health-related quality of life (HRQoL) impacts. This systematic review synthesizes global evidence on the economic and HRQoL consequences of PTSD to inform policy decision-making.

**Methods:**

A systematic search of six databases (Web of Science, EMBASE, MEDLINE, PsycINFO, PubMed, and EconLit) was conducted in April 2024 and updated in April 2025. Studies reporting real-world costs or HRQoL outcomes in PTSD populations were included. Quality was assessed using the Joanna Briggs Institute critical appraisal tools. Data were synthesized narratively due to heterogeneity in study design, populations, and outcome measures.

**Results:**

Twenty studies met inclusion criteria, spanning 2000–2024 and representing diverse settings including the US, Europe, South Korea, and Australia. However, most were from high-income countries, with limited representation from low- and middle-income countries (LMICs). PTSD was associated with high direct healthcare costs (e.g., $66.0 billion in US civilians) and substantial indirect costs from lost productivity, disability, caregiving, and premature mortality. Veterans and conflict-affected populations bore particularly high economic burdens. HRQoL was markedly reduced across all populations studied, with EQ-5D, AQoL, and MANSA scores indicating significant functional and psychosocial impairment. Co-occurring conditions (e.g., depression, anxiety), trauma severity, and sleep disturbances were key drivers of reduced HRQoL.

**Conclusions:**

PTSD imposes a substantial economic and quality of life burden globally. Findings provide evidence supporting the need for comprehensive treatment approaches, long-term support systems, and policies that address both economic and psychosocial outcomes in PTSD care.

**Supplementary Information:**

The online version contains supplementary material available at 10.1186/s13561-026-00800-5.

## Introduction

Post-traumatic stress disorder (PTSD) is a chronic and debilitating psychiatric condition triggered by exposure to severe traumatic events, such as accidents, sexual violence, natural disasters, and interpersonal violence [1]. Characterized by persistent re-experiencing of traumatic events, avoidance behaviors, hyperarousal symptoms, and negative alterations in mood and cognition, PTSD profoundly impacts individuals’ emotional, social, and physical well-being [2]. The World Mental Health Survey reported a global lifetime prevalence of PTSD of approximately 3.9% in the general population and 5.6% among individuals exposed to trauma [3]. Epidemiological studies suggest significant global variability in PTSD prevalence, ranging from approximately 1.1% to 9.2%, reflecting the diverse nature of traumatic exposures and cultural differences in recognizing and reporting symptoms [4, 5]. The chronicity and severity of PTSD are often exacerbated by comorbid psychiatric disorders such as anxiety, depression, and substance use, complicating clinical management and exacerbating overall patient burden [6]. The multifaceted consequences of PTSD extend well beyond the individual, exerting extensive pressure on healthcare systems, economic productivity, and societal resources. Individuals with PTSD experience frequent hospitalizations, increased healthcare utilization, and elevated medication and psychotherapy costs compared to non-affected individuals [7]. Beyond direct medical expenses, PTSD significantly contributes to indirect economic burdens through lost productivity, disability, unemployment, absenteeism, involvement with the criminal justice system, stigma, and early retirement [8, 9]. For instance, in the United States, total excess economic costs of PTSD were recently estimated at approximately $232 billion annually, highlighting its substantial economic impact from both civilian and military perspectives [10]. Such high economic burdens are not unique to the United States; similar patterns have been reported globally, including Australia, Germany, South Korea, Canada, and regions affected by prolonged conflicts such as Northern Ireland and former Yugoslavia [11–16]. Simultaneously, the impact of PTSD on quality of life (QoL) has emerged as a critical area of investigation. Health-related quality of life (HRQoL), encompassing mental, physical, and social functioning domains, is notably impaired in PTSD populations [17]. The multidimensional nature of QoL deficits includes significant decreases in general health perception, daily functional impairment, and compromised interpersonal relationships [17, 18]. Consistently, studies report substantial decrements in QoL among PTSD patients, often exacerbated by co-occurring disorders such as depression and anxiety, and further compounded by factors such as trauma severity, sleep disturbances, and functional impairments in social and occupational domains [19, 20].

Despite the accumulating evidence, a comprehensive synthesis integrating economic and QoL outcomes remains limited. Existing reviews often focus either exclusively on economic costs or exclusively on QoL impacts, with insufficient integration of these dimensions [7, 17]. Moreover, heterogeneity in study designs, measurement tools, and population characteristics complicates meaningful synthesis and generalization of findings, thereby limiting informed policy decisions and clinical practice guidelines. Because both costs and HRQoL feature in evaluations of interventions aimed at managing PTSD, it is important that both are examined together. Given these critical needs, this systematic review aims to synthesize PTSD’s economic and HRQoL impacts to inform clinicians, policymakers, and researchers and to support resource allocation and targeted intervention planning.

## Methods

A protocol was developed and registered with International Prospective Register of Systematic Reviews (PROSPERO, #CRD42023425272) prior to the conduct of this review. The report adheres to PRISMA (Preferred Reporting Items for Systematic Reviews and Meta-Analyses) guidelines (complete PRISMA checklist is provided in Supplementary materials).

### Search strategies and inclusion criteria

Inclusion criteria for studies were based on the PICOS framework (Population, intervention/exposure, comparator, outcomes, and study design) as follows: (1) Population: individuals with diagnosed PTSD regardless of treatment status or source of trauma; no age restrictions were applied; (2) Intervention/exposure: any PTSD treatment, care pathway, or observed PTSD population; (3) Comparator: studies were eligible if they included a non-PTSD comparator group, a pre/post comparison, a population norm, or another usable reference benchmark that allowed the economic or HRQoL burden associated with PTSD to be interpreted; studies without a usable comparator or reference benchmark were excluded; (4) Outcomes: two main outcomes were cost and HRQoL, including preference-based utility measures (utilities, quality-adjusted life-years [QALYs], and/or disability-adjusted life-years [DALYs]) and validated HRQoL instruments (e.g., EuroQol 5-Dimension [EQ-5D], Assessment of Quality of Life [AQoL], and the Manchester Short Assessment of Quality of Life [MANSA]); and (5) Study design: observational studies using real-world data. We excluded systematic reviews, letters, commentaries, modelling studies without real-world data, conference proceedings without full text, abstract-only reports, and study protocols (Details of inclusion and exclusion criteria are provided in Supplementary materials). An individual is considered to have PTSD if it was diagnosed and marked by ICD-9 (309.81) or ICD-10 (F43.1) or equivalent in the patient’s medical record.

Six databases including Web of Science, EMBASE, MEDLINE, PsycINFO, PubMed, and Econlit were searched in April 2024 and updated in April 2025. Searches had no date restrictions but were limited to English-language publications. (Detailed search strategies for all databases are provided in Supplementary materials).

### Data collection and analysis

All citations resulting from the searches were imported into Covidence. After removing duplicated citations, the selection process was conducted in three stages including (1) Title and abstract screening; (2) Full text review; and (3) Quality assessment using Joanna Briggs Institute (JBI) critical appraisal checklists for cohort studies (*n* = 16) and cross-sectional studies (*n* = 4). The risk of bias was ranked as high, medium, and low (Ratings for all included studies are provided in Supplementary materials). Studies rated as high risk of bias would be excluded. Each stage was conducted independently by two reviewers (Stage 1: TTN and MS; stage 2 and 3: THT and MXT). Conflicts in the include/exclude decision were resolved by reaching consensus among two reviewers or consulting a third reviewer (CON) if agreement could not be reached.

A data extraction form was created on Covidence platform and pilot tested on four randomly included studies (~ 20% of all included studies) and refined accordingly. Extracted data included general information (title, author, year of publication, and country in which the study was conducted), methods (aims, study design, and source of data), participants’ characteristics (description, inclusion/exclusion criteria, sample size), interventions/comparators, outcomes (HRQoL and cost). Two reviewers extracted the data (THT and MXT). Each completed the extraction of 50% of the included studies by themselves and then reviewed the extraction of the other.

To improve descriptive comparability across countries and years, reported monetary values were standardized to 2024 US dollars using purchasing power parity conversion and inflation adjustment with the CCEMG-EPPI Centre Cost Converter. These standardized values were used for descriptive comparison only and do not eliminate differences across studies in costing perspective, cost components, healthcare systems, or patient case mix.

Findings from included studies were organized and presented (narrative synthesis) by pre-determined outcomes. Narrative synthesis was chosen because of the high heterogeneities in reported costs and QALYs of PTSD due to different methods, sources of trauma, and participants. Population subgroup analysis was also provided where possible.

## Results

### Included studies

**Fig. 1:**
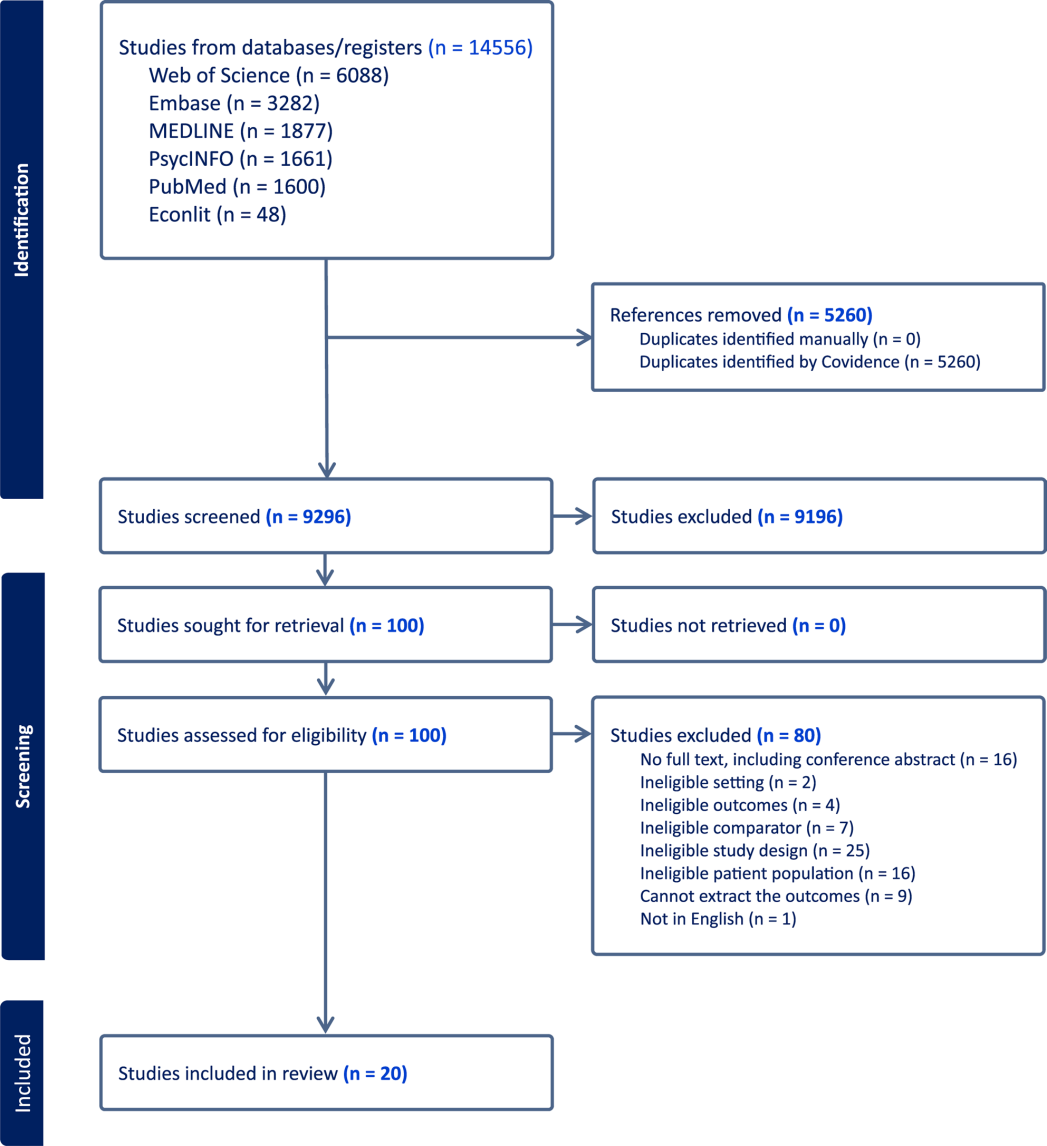
PRISMA flowchart

A total of 14,556 records were identified through database searches, including Web of Science (*n* = 6,088), Embase (*n* = 3,282), MEDLINE (*n* = 1,877), PsycINFO (*n* = 1,661), PubMed (*n* = 1,600), and EconLit (*n* = 48). Following the removal of 5,260 duplicates using Covidence, 9,296 records were screened based on titles and abstracts. Of these, 9,196 records were excluded for not meeting the inclusion criteria. One hundred studies were sought for retrieval and assessed for eligibility. During the eligibility assessment, 80 studies were excluded for various reasons, including no full text (including conference abstract) (*n* = 16), non-English language publications (*n* = 1), ineligible setting (*n* = 2), ineligible outcomes (*n* = 4), no usable comparator or reference benchmark (*n* = 7), ineligible study design (*n* = 23), ineligible patient population (*n* = 16), inability to extract relevant outcomes (*n* = 9). Ultimately, 20 studies were included in this systematic review, with publication years ranging from 2000 to 2024. (Fig. 1. PRISMA flowchart).

The included studies predominantly utilized cohort design (16/20), complemented by cross-sectional analyses (4/20). Geographically, the studies were distributed primarily across the United States (9/20) [10, 21–28], followed by Australia (3/20) [11, 29, 30], Germany (2/20) [15, 31], South Korea (2/20) [14, 32], Northern Ireland (1/20) [13], Canada (1/20) [16], and Italy (1/20) [33], and former Yugoslavian countries including Serbia, Croatia, and Bosnia-Herzegovina (1/20) [12].

### Study population characteristics

Among the 20 studies, 5 explicitly reported mean age characteristics or age-related patterns. Older adult populations with a mean age exceeding 60 years were explicitly noted in veteran studies [11, 26, 27]. In contrast, younger populations were investigated in two studies focusing on adolescents and young adults experiencing sexual trauma or abuse [15, 30]. Gender characteristics were explicitly described in 5 of the 20 studies, primarily reflecting male-dominated populations within veteran studies [11, 23, 26, 27] and female predominance in civilian and hospital settings [32].

The predominant trauma sources comprised military combat reported in seven included studies [10, 11, 13, 23, 24, 26, 27], interpersonal trauma due to sexual abuse in two studies [15, 30], motor vehicle accidents in one study [29], and medical illness in three studies [28, 31, 33]. Additionally, North Korean defectors represented a distinct subgroup in one study [14]. Trauma source was not reported in the remaining studies. For clarity, studies are summarized by outcome: articles reporting economic outcomes are presented in Table 1, and articles reporting HRQoL outcomes are presented in Table 2; one study contributed to both tables when both outcomes were reported.

**Table 1:** A summary of the indicators of economic burden of PTSD in included articles (*n* = 18) adjusted for purchasing power parity conversion and inflation adjustment rates for 2024, based on International Monetary Fund through a tool developed with support from the Cochrane group [34]

No.	Author and Year	Country	Country Group	Research Design	Calculation Method	Method	Cost Perspective	Population	Source of Data	Indicator of Calculated Disease Burden	Total Economic Loss	Direct Cost	Indirect Cost	Adjusted to PPP and inflation
1	Marshall 2000 [11]	Australia	High income	Retrospective Cohort	Health Expenditure	Prevalence-based	Healthcare system	*N* = 641 Australian Vietnam veterans	Department of Veterans’ Affairs and the Health Insurance Commission	Direct medical cost		$226 per patient (USD 1999)		399.5
2	Marciniak 2005 [21]	United States	High income	Retrospective Cohort	Health Expenditure	Prevalence-based	Healthcare system	*N* = 6,497 Privately insured individuals	MarketScan Databases	Direct medical cost		$3,939.58 per patient (USD 1999)		6,965.11
3	O’Donnell 2005 [29]	Australia	High income	Retrospective Cohort	Health Expenditure	Prevalence-based	Healthcare system	*N* = 255 Motor vehicle accidents survivors	Longitudinal study of psychiatric outcomes following severe injury	Direct medical cost		$47,133 per patient (USD 2001)		79,688.29
4	François 2010 [22]	United States	High income	Retrospective Cohort	Health Expenditure	Prevalence-based	Healthcare system	*N* = 3,719 Patients aged 18 years or older	PharMetrics database	Direct medical cost		$8,383 per patient (USD 2005)		12,922.64
5	Priebe 2010 [12]	Serbia, Croatia, and Bosnia-Herzegovina		Prospective Cohort	Health expenditure	Incidence-based	Healthcare system	*N* = 325 War-related PTSD adults	Four specialized centers in three countries, Bosnia-Herzegovina (Sarajevo), Croatia (Rijeka and Zagreb), and Serbia (Belgrade)	Direct medical cost		€307 (2004–2005 Euros)		143.15*
6	Patterson 2011 [27]	United States	High income	Retrospective Cohort	Health Expenditure	Prevalence-based	Healthcare system	*N* = 70 Veterans	An overseas federal health care facility	Direct medical cost		Acute symptom individual: $19,527 per patient Chronic symptom individual: $28,292 per patient		Acute: 26,832.62; Chronic: 38,876.86
7	Meyers 2013 [23]	United States	High income	Retrospective Cohort	Health Expenditure	Prevalence-based	Healthcare system	*N* = 70 Veterans	National VA databases	Direct medical cost		Before treatment: $5,173.20 per patient After treatment: $3,133.10 per patient		Before: 6,858.05 After: 4,153.51
8	Ferry 2015 [13]	Northern Ireland	High income	Cross-sectional	Cost of illness	Prevalence-based, human capital approach	Society	*N* = 1,986 Adults aged 18 and over	Northern Ireland Study of Health and Stress	Direct medical cost and economic loss	Total: £139,780,472 (2008)	£32,975,590 (2008)		Total: 296,287,043.55 Direct: 69,897,031.61
9	Haviland 2016 [24]	United States	High income	Retrospective Cohort	Health Expenditure	Prevalence-based	Healthcare system	*N* = 1,477,944 discharges from individuals aged 20 and older with PTSD diagnosis	Nationwide Inpatient Sample (NIS) database	Direct medical cost		Total: $34.9 billion (2011) Men: $21,614- $35,818 Women: $18,870- $33,291		Total: 34.9 Men: 29,700.42-49,218.55 Women: 25,929.81-45,746.13
10	Noh 2019 [14]	South Korea	High income	Retrospective Cohort	Health expenditure	Prevalence-based	Healthcare system	*N* = 3,974 North Korean defectors	Electronic medical records	Direct medical cost	953.6 USD (2016)	Not reported	Not reported	1,216.48
11	Bothe 2020 [15]	Germany	High income	Retrospective Cohort	Cost of illness	Incidence-based	Healthcare system	*N* = 12,887 newly diagnosed PTSD patients	Nationwide InGef research database	Total economic loss	42,870 EUR (2010–2017)	Not reported	Not reported	77,589.21
12	von der Warth 2020 [31]	Germany	High income	Retrospective Cohort	Cost of illness	Incidence-based	Healthcare system	*N* = 219 inpatients with PTSD	German university hospital records	Direct medical cost	€7919 per patient (2014)	€2311 additional hospitalization cost	€1387 additional reimbursement	Total: 13,450.61 Direct: 3,925.29 Indirect: 2,355.85
13	Kim 2021 [32]	South Korea	High income	Retrospective Cohort	Health expenditure	Prevalence-based	Healthcare system	*N* = 21,402 PTSD patients	National Health Insurance Service Database	Direct medical cost	3,911,283 USD (2017)	310–426 USD per person annually (2017). Most cost from outpatient visits & psychiatric department inpatient units	Not reported	4,896,499.82; 388.09-533.31
14	Roggenkamp 2021 [16]	Canada	High income	Prospective Cohort	Health expenditure	Prevalence-based	Healthcare system	*N* = 41 PTSD patients receiving ISTDP	Centre for Emotions and Health (CEH) at Dalhousie University database	Direct medical cost reduction	Not reported	Statistically significant reduction in physician costs (from 800 to 584 Canadian dollars in 2007) and hospital cost (from 4639 to 781)	Not reported	Physician: 959.28-700.28; Hospital cost: 5,562.65–936.50
15	Davis 2022 [10]	United States	High income	Cross-sectional	Cost of illness	Prevalence-based, human capital approach	Society	N=total U.S. PTSD population	MarketScan, U.S. Census, VA, DOD, governmental agencies	Total economic burden	Overall (US, 2018): Total economic burden $232.2B; per-person excess cost $19,630 Civilian: Total $189.5B; per-person excess cost $18,640. Military: Total $42.7B; per-person excess cost $25,684.	66 billion USD (direct medical costs); direct healthcare costs per individual of $6,495 (civilian) and $6,071 (military); direct non-healthcare costs per individual of $3,021	42.7 billion USD (unemployment, disability, mortality); per-individual indirect costs: Civilian (per person): Unemployment $4,196; productivity loss $2,875; caregiving $3,274; premature mortality $132. Military (per person): Unemployment $2,127; productivity loss $3,374; caregiving $2,062; premature mortality $749.	Total economic burden: 283.87B Civilian total: 231.66B Military total: 52.2B
16	Harper 2022 [26]	United States	High income	Prospective Cohort	Health expenditure	Incidence-based	Healthcare system	*N* = 1,377 veterans	Veterans Health Administration (VHA) data	Direct medical cost	Increased healthcare costs for PTSD veterans compared to non-PTSD veterans	9,426.63 USD per patient (2013–2015)	Not reported	12,496.76
17	Lorenzoni 2024 [33]	Italy	High income	Cross-sectional	Cost of illness	Prevalence-based	Society	*N* = 301 systemic autoimmune disease patients	Specialized outpatient clinic in Tuscany, Italy	Direct and indirect cost	3,670 EUR per PTSD patient annually	1,193 EUR (non-healthcare cost)	3,741 EUR (indirect cost)	5,227.92 per patient; 1,699.43 non healthcare, indirect cost 5,329.06
18	Stanicic 2024 [28]	United States	High income	Retrospective Cohort	Health expenditure	Incidence-based	Healthcare system	*N* = 5076 patients	Merative MarketScan Commercial and Medicare Part B claims database	Direct medical cost	Not reported	First-year follow-up cost: prescription cost ($1,679), outpatient cost ($4,140), inpatient cost ($1,360), ED cost ($536), PTSD total cost ($1,339), All-cause total cost ($7,714)	Not reported	Prescription: 1,717.03 Outpatient: 4,233.78 Inpatient: 1,390.81 ED: 548.14 PTSD total cost: 1,369.33 All-cause total cost: 7,888.75

*Considered Croatia for PPP and inflation

**Table 2:** A summary of the quality of life burden of PTSD in included articles (*n* = 3)

No	Authors and Year	Country	Country Group	Study Design	Population	Data Source	Quality of Life Questionnaire	Quality of Life Impact
1	Priebe 2010 [12]	Bosnia-Herzegovina, Croatia, Serbia	Upper middle income	Prospective Cohort	*N* = 526 war-related PTSD patients	Specialized PTSD treatment centers	MANSA	Small but significant improvements in quality of life after 12 months of treatment. MANSA scores at baseline and follow-up were both below the neutral midpoint of 4, indicating overall dissatisfaction with life
2	Gospodarevskaya 2013 [30]	Australia	High income	Cross-sectional	*N* = 993 Adolescents and young adults with sexual abuse history	2007 Australian National Survey of Mental Health	AQoL	Significant loss of HRQoL (mean utility score: 0.68; with comorbid depression: 0.61, population norm: 0.87)
3	Le 2018 [25]	United States	High income	Doubly randomized preference trial	*N* = 200 PTSD patients	Clinical trial dataset	EQ-5D-3 L	PE therapy improved HRQoL (increase in EQ-5D scores by 0.150, *p* = 0.025)

### Overall economic burden of PTSD

Comprehensive global data on the economic burden of PTSD remains limited. The economic burden of PTSD in the United States is substantial. In 2018, the total annual excess economic burden was estimated at $232.2 billion [10]. Of this total, $189.5 billion (81.6%) was attributed to the civilian population, while $42.7 billion (18.4%) was associated with the military (veteran and active-duty) population [10]. The average excess cost per individual with PTSD was $19,630, with $18,640 for civilians and $25,684 for the military [10]. These figures were calculated from a societal perspective. For the civilian population, the primary drivers of this economic burden were direct health care costs ($66.0 billion) and unemployment costs ($42.7 billion). In contrast, for the military population, the excess burden was mainly due to disability costs ($17.8 billion) and direct health care costs ($10.1 billion) [10]. The total direct and indirect cost of PTSD in Northern Ireland was estimated to be nearly £172.8 billion in 2008 [13]. This included direct costs of £33.0 million and indirect costs of £139.8 million [13]. The significantly higher indirect costs in Northern Ireland suggest a substantial impact of PTSD on productivity and other societal factors beyond direct healthcare expenses, potentially reflecting the long-term consequences of conflict-related trauma in the region. A study conducted in Italy between 2021 and 2022 found that the overall costs for patients with systemic autoimmune disease (SAD) who also had PTSD were EUR 3,670 per patient per year, compared to EUR 2,736.7 per patient per year for SAD patients without PTSD [33]. The presence of PTSD was estimated to increase overall costs, direct non-healthcare costs, and indirect costs in individuals with SAD [33]. In Korea, the total direct medical cost of PTSD showed a continuous increase between 2011 and 2017, reaching 426 USD per individual [32]. The majority of these direct medical costs were claimed by outpatient clinics and psychiatric department inpatient units. Studies in Australia have indicated that medical costs for Vietnam veterans with PTSD were 60% higher than average [11].

### Direct costs and healthcare utilization

Direct healthcare costs associated with PTSD encompass a range of services, including medical care, hospitalization, medication, and mental health services such as psychotherapy and counseling. In the United States, these costs amounted to $66.0 billion for the civilian population and $10.1 billion for the military population in 2018, corresponding to excess direct healthcare costs of approximately $6,495 per civilian and $6,071 per military individual with PTSD [10, 26]. In Korea, the majority of direct medical costs for PTSD were associated with outpatient clinics and psychiatric department inpatient units. In Australia, among Vietnam veterans, Marshall et al. reported direct medical cost of approximately $226 per patient with PTSD (1999 USD) [11]. In Korea, a nationwide claims study reported annual direct medical cost per person with PTSD of $310–$426 (2017) (total $3.91 million across 21,402 patients) [32], and among North Korean defectors the mean direct medical cost was $953.6 per patient (2016) [14]. Individuals with PTSD tend to have increased healthcare utilization compared to those without the disorder. This includes higher rates of psychiatric contact, as well as increased utilization of both inpatient and outpatient treatment services [26, 28]. Studies have also shown an increased use of psychotropic medications among individuals with PTSD [26]. In the US, data from 2002 to 2011 indicated higher rates of PTSD-related hospitalizations, particularly among women aged 20 to 44 [24]. Furthermore, two studies in South Korea found that those with PTSD had a higher average number of outpatient visits and utilized a greater variety of medical services compared to those without PTSD [14].

### Indirect costs

Indirect costs associated with PTSD significantly contribute to its economic burden. These costs arise from lost productivity due to unemployment, absenteeism (days off work), presenteeism (reduced productivity while at work), and educational interruption, disability costs, premature mortality costs, and caregiving costs [10, 31, 33]. In the US in 2018, lost productivity costs were estimated at $74.7 billion for the civilian population and $9.1 billion for the military population [10]. At the individual level, this corresponds to approximately $4,196 (civilian) and $2,127 (military) for unemployment and $2,875 (civilian) and $3,374 (military) for on-the-job productivity loss (absenteeism + presenteeism) [9]. These figures underscore the long-term impact of PTSD on the ability of military personnel and veterans to function and work, often leading to long-term disability benefits and reduced economic participation. PTSD is associated with an increased risk of premature mortality, which also contributes to the economic burden. In the US in 2018, costs associated with premature mortality were estimated at $1.3 billion for civilians and $1.2 billion for the military [9]. Per individual with PTSD, premature-mortality costs were about $132 (civilian) and $749 (military) [9]. Notably, the annual all-cause mortality rate for individuals with PTSD in the military/veteran population was estimated to be 80% higher than for those without PTSD, resulting in approximately $1 billion in excess costs annually. In the US in 2018, caregiving costs related to PTSD were estimated at $33.3 billion for the civilian population and $3.4 billion for the military population [10]. Per individual with PTSD, caregiving costs were approximately $3,274 (civilian) and $2,062 (military) [9]. This substantial financial impact on caregivers underscores the wider societal costs of PTSD, extending beyond those directly affected by the disorder.

### Economic burden on specific subgroups

#### Veterans and military personnel

Veterans and active military personnel experience a disproportionately high economic burden associated with PTSD. In the US, the per-person cost of PTSD is higher for military populations compared to civilians, with significant costs attributed to disability. Prevalence rates of PTSD are also higher in this population compared to the general public. For instance, the economic burden of PTSD and depression among Operation Enduring Freedom/Operation Iraqi Freedom veterans alone was estimated at $4–6 billion over a two-year period, including medical care, lost productivity, and lives lost through suicide. These figures highlight the substantial and long-lasting economic consequences of combat exposure and service-related trauma [10].

### Quality of life burden

Across the 20 included studies, various validated instruments were used to assess health-related quality of life (HRQoL) in PTSD populations. These included the EuroQol-5 Dimensions 3-level (EQ-5D-3 L) questionnaire [25], Manchester Short Assessment of Quality of Life (MANSA [12], and Assessment of Quality of Life (AQoL) questionnaire [30].

### Quality of life impact

Le et al. used EQ-5D-3 L to evaluate outcomes among U.S. patients undergoing different PTSD treatments. Their findings showed that Prolonged Exposure (PE) therapy was significantly associated with improved HRQoL scores, especially among patients who expressed a preference for this specific treatment [25]. Priebe et al. used MANSA to assess HRQoL among adults with PTSD in Bosnia-Herzegovina, Croatia, and Serbia, finding modest yet statistically significant improvements in HRQoL after twelve months of targeted PTSD treatment at specialized centers [12]. Gospodarevskaya applied the AQoL tool in a study of Australian adolescents and young adults with PTSD following sexual abuse, reporting an average utility score of 0.68 compared to the age-adjusted population norm of 0.87 [30]. This ~ 0.19 utility deficit is comparable in magnitude to the EQ-5D decrement associated with heart failure (≈ 0.185) in contemporary UK data [35]. For those explicitly identifying sexual abuse as their most traumatic event, utility scores were even lower at 0.61 [30]. The successful treatment of PTSD in these children was estimated to result in savings of approximately 2.05 quality-adjusted life years (QALYs) per treated individual [30].

### Factors driving quality of life impact

Several studies identified key factors associated with lower HRQoL scores. Anxiety and depression consistently appeared as critical contributing factors to reduced HRQoL, as reported by Bothe et al. [15]. In their analysis, the presence and severity of these co-occurring mental health disorders directly correlated with worsened QoL outcomes, especially in younger populations suffering trauma from abuse [15]. Sleep disturbances were also identified as notable contributors to impaired QoL in PTSD. Specifically, Gospodarevskaya noted that among sexually abused Australian children, sleep disruption significantly impacted QoL - sometimes even surpassing the detrimental effects associated directly with PTSD symptom severity [30].

### Quality assessment

Overall, the included studies met most JBI critical appraisal criteria and were judged to have low risk of bias. Among the cohort studies (*n* = 16), checklist adherence ranged from 90.9% to full item fulfillment, and all were categorized as low risk of bias. The four cross-sectional studies also met all applicable JBI checklist items and were categorized as low risk of bias. These ratings indicate generally strong methodological quality based on the JBI criteria, but they should not be interpreted as evidence that the studies were free from bias.

## Discussion

This review provides an overview of the cost and impact on Qol among individuals with PTSD. It highlights the considerable variability in the magnitude of impact related in part to the source of PTSD, the healthcare system in which it us observed and likely the priority afforded mental health and particular patient groups within that system. Differences in methods used and the analytic perspective adopted also contribute to differences and complicate comparative analysis across studies. The predominance of studies from developed countries – particularly from a small number of Western countries – is notable. It highlights a gap in data from low- and middle-income countries (LMICs) which is especially concerning given the prevalence of conflict in many of these settings. In such contexts, the burden of PTSD may not only be under studied but also underreported, as immediate demands on services, population displacement, and the challenges of data collection in war zones make research difficult [36]. In many regions affected by ongoing and protracted conflicts – such as Palestine, Sudan, Myanmar, Libya, and Yemen – the true burden of PTSD is especially difficult to measure. Large-scale population displacement, limited healthcare infrastructure, and the practical and ethical challenges of conducting research in active conflict zones all contribute to major gaps in data. As a result, the prevalence and consequences of PTSD in these settings are likely underestimated, further widening the evidence gap between high‑income and conflict‑affected countries.

Standardized to 2024 PPP-adjusted US dollars, the reported economic burden of PTSD appeared highest in the United States, with excess per-person costs of $23,998 in the societal analysis by Davis et al. [10]. Cost estimates from other settings were also substantial, including mean healthcare costs of 13,450 PPP-adjusted US dollars in Germany [31] and 5,228 PPP-adjusted US dollars in Italy among patients with autoimmune disease and comorbid PTSD [33]. However, these figures should be interpreted cautiously. PPP and inflation adjustments improve descriptive comparability across currencies and years, but they do not resolve heterogeneity in study populations, trauma source, comparator structure, costing perspective, or the specific cost components included.

In the US, while civilians account for 82% of total PTSD economic burden [10], the average excess cost per individual with PTSD remains substantially higher for veterans compared to civilians and has risen faster over time, increasing from $25,684 in 2018 to an inflation-adjusted $31,399 in 2024, compared with a rise from $18,640 to approximately $22,787 for civilians [10]. Similarly, it is 60% higher for veterans than the average across all PTSD patients in Australia [11]. The higher costs for veterans may be due to greater functional impairment but likely also reflects a higher priority afforded this group in government insurance systems. This contrasts with civilians who have less public coverage and rely on private insurance and out-of-pocket expenses to a greater extent. Differences in cost patterns may also reflect how recognition of PTSD has evolved unevenly across populations. Historically, PTSD in military personnel – previously termed “shell shock” – was acknowledged as early as the First World War, leading to earlier development of dedicated services for veterans [37]. In contrast, PTSD related to other forms of trauma, such as childbirth or partner trauma in non-birthing parents, has only been more widely recognized in recent years. This uneven timeline in diagnosis and service development likely contributes to disparities in access to care, support structures, and associated costs across different groups.

Our review highlights substantial direct healthcare costs associated with PTSD, including medical care, hospitalization, medication, and mental health services. In the US, these costs are high ($66 billion for civilians, $10.1 billion for the military), while estimates from Germany [31] and Korea [32] reflect more context-specific costs such as those arising after abuse or within inpatient and outpatient psychiatric settings. Interestingly, several important cost components were not reported in the included studies. These include direct non-healthcare costs such as research and training, substance use treatment, psychotherapy not covered by insurance, homelessness, disability support, and interactions with the criminal justice system – factors likely to further increase the true economic burden of PTSD.

Indirect costs, including productivity losses, caregiving demands, and disability-related expenses also contribute significantly. The substantial productivity losses reported among young adults [38] and both civilian and military groups [10], underscore how PTSD can disrupt not only individual lives but also workforce participation and efficiency, disruptions that may have lifelong effects. The substantial caregiving and disability costs further illustrate the wider societal impact of PTSD that extends well beyond direct medical expenses, affecting families and community. However, it is worth noting that disability costs should be interpreted with caution as these represent financial commitment from governments and viewed as a redistribution of resources rather than a pure economic loss.

Individuals with PTSD tend to use more healthcare services than those without the condition, including higher rates of hospitalizations, more frequent outpatients’ visits and broader use of medical services [24, 28]. This increased utilization retains even after accounting for comorbidities, and is particularly pronounced among women [28]. PTSD plays a critical role in driving this demand. It mediates the relationship between trauma exposure and mental health service use and often serves as the main factor behind long-term healthcare needs – not just for psychological care, but also physical health conditions that may be worsened by PTSD. For example, in the Korean study [32], patients with PTSD had significantly more outpatient visits and used a wider variety of services than their non-PTSD counterparts. As PTSD becomes more widely recognized and diagnosed, particularly beyond traditional populations like veterans, demand for services and associated costs are likely to rise. These trends reflect both the clinical complexity and economic impact of PTSD and highlight the urgent need for targeted, accessible interventions. Investing in early and effective treatment may help reduce the long-term burden on healthcare systems.

PTSD is associated with reduced HRQoL across a wide range of populations and settings. Several factors that contribute to poorer QoL outcomes, include co-occurring anxiety and depression, sleep difficulties, and the severity of trauma exposure [17, 39, 40]. These findings suggest that effective PTSD care must go beyond symptom reduction and address broader areas such as sleep, social functioning, and emotional well-being. Improvements in QoL following certain interventions, such as PE, also highlight the value of patient-centered treatment tailored to individual preferences. Notably, the utility score for young people with PTSD and comorbid depression has been reported as low as 0.68, compared to a general population norm of 0.87 [30]. This level of impairment is comparable to that seen in people with stroke (mean EQ-5D score of 0.65) [41] and greater than that typically reported in individuals with type 2 diabetes (mean EQ-5D score of 0.80) [35]. These comparisons illustrate the severity of PTSD’s impact on daily functioning, and, when combined with variations in healthcare access and societal support across countries, help explain the heterogeneity in both economic and health outcomes globally.

Overall, this review highlights the complexity of quantifying PTSD’s economic burden due to substantial heterogeneity in study population, settings, and cost components. Studies vary from hospital-based cost estimates to community-level analyses and from populations with fewer comorbidities (e.g., children and adolescents) to those with more complex clinical profiles (e.g., veterans and older adults). Despite these limitations and the potential underestimation, the findings remain valuable due to the methodological rigor of the included studies.

Beyond methodological gaps, important trauma contexts remain underrepresented in the literature, including childhood abuse, obstetric trauma, and trauma linked to displacement, systemic violence, or neglect. This imbalance likely reflects where research infrastructure, data availability, and funding have historically been concentrated. As a result, the existing evidence base may not fully capture the economic and HRQoL burden of PTSD across the full range of trauma-exposed populations, particularly in low-resource and conflict-affected settings. Addressing these gaps should be a priority for future research to improve the completeness and generalizability of the evidence.

This review has some limitations. First, substantial heterogeneity was observed across the included studies in terms of design, populations, cost estimation methods, and HRQoL measurement tools. This variability hinders quantitative synthesis and limited direct comparisons across settings and populations could be made. Second, most studies were conducted in high-income countries, particularly the United States, with limited representation from LMICs. As a result, the findings may not fully capture the global burden of PTSD, particularly in regions where conflict, displacement, and trauma are widespread but under-researched. Third, the review was restricted to English-language publications, which may have led to the exclusion of relevant studies published in other languages, introducing potential language bias. Fourth, our requirement for an ICD-coded PTSD diagnosis and for a usable comparator or reference benchmark may have excluded relevant evidence relying on questionnaire-based PTSD measures or single-arm burden estimates, potentially contributing to the limited HRQoL evidence identified and under-representation of certain trauma types and LMIC settings. Finally, four of the included studies used cross-sectional designs, limiting the ability to investigate causal relationships between PTSD and economic or quality-of-life outcomes.

## Conclusions

This review highlights the substantial global burden of PTSD on both economic resources and individuals’ quality of life. The evidence shows that PTSD leads to high direct and indirect costs, including healthcare expenses, lost productivity, and caregiving demands, while also severely impacting daily functioning, well-being, and social participation. These findings point to the importance of integrated approaches that combine effective clinical treatment with broader social and economic support.

To strengthen future research and improve comparability across studies, there is a clear need for standardized methodologies and reporting practices. These should account for variations in population characteristics, care settings, and trauma types, while also capturing the full range of PTSD-related costs and quality-of-life impacts. Such efforts are essential for informing policy, guiding resource allocation, and improving outcomes for people living with PTSD.

## Supplementary Information

Supplementary Material 1.

Supplementary Material 2.

Supplementary Material 3.

## Data Availability

All data generated or analysed during this study are included in this published article and supplementary materials.
