# Supplementary material for: Economic and health-related quality of life impacts of posttraumatic stress disorder: a systematic review
Source: Health Econ Rev. 2026 Jun 2;16:93. doi: 10.1186/s13561-026-00800-5 (PMC13463949; doi:10.1186/s13561-026-00800-5)
Supplement: Supplementary file 2 [file 13561_2026_800_MOESM2_ESM.docx]

| Study (Year) | Q1^a^ | Q2 | Q3 | Q4 | Q5 | Q6 | Q7 | Q8 | Q9 | Q10 | Q11 | % Yes | Risk^b^ |
| --- | --- | --- | --- | --- | --- | --- | --- | --- | --- | --- | --- | --- | --- |
| Marshall 2000 | Y | Y | Y | Y | Y | Y | Y | Y | Y | N/A | Y | 90.9% | Low |
| Marciniak 2005 | Y | Y | Y | Y | Y | Y | Y | Y | Y | N/A | Y | 90.9% | Low |
| O’Donnell 2005 | Y | Y | Y | Y | Y | Y | Y | Y | Y | N/A | Y | 90.9% | Low |
| François 2010 | Y | Y | Y | Y | Y | Y | Y | Y | Y | N/A | Y | 90.9% | Low |
| Priebe 2010 | Y | Y | Y | Y | Y | Y | Y | Y | Y | N/A | Y | 90.9% | Low |
| Patterson 2011 | Y | Y | Y | Y | Y | Y | Y | Y | Y | N/A | Y | 90.9% | Low |
| Meyers 2013 | Y | Y | Y | Y | Y | Y | Y | Y | Y | N/A | Y | 90.9% | Low |
| Haviland 2016 | Y | Y | Y | Y | Y | Y | Y | Y | Y | N/A | Y | 90.9% | Low |
| Bothe 2020 | Y | Y | Y | Y | Y | Y | Y | Y | Y | N/A | Y | 90.9% | Low |
| Von der Warth 2020 | Y | Y | Y | Y | Y | Y | Y | Y | Y | N/A | Y | 90.9% | Low |
| Kim 2021 | Y | Y | Y | Y | Y | Y | Y | Y | Y | N/A | Y | 90.9% | Low |
| Harper 2022 | Y | Y | Y | Y | Y | Y | Y | Y | Y | N/A | Y | 90.9% | Low |
| Stanicic 2024 | Y | Y | Y | Y | Y | Y | Y | Y | Y | N/A | Y | 90.9% | Low |

^a^Q1 – Q11 indicate questions 1 to 11 based on the JBI critical appraisal checklist for cohort studies.

^b^The risk of bias was ranked as high when the study reached up to 49% of “yes” scores, moderate when the study reached from 50 to 69% of “yes” scores, and low when the study reached more than 70% of “yes” scores. ‘Y’ indicates yes, ‘N’ indicates no, ‘U’ indicates unclear and ‘N/A’ indicates unclear not applicable.

| Study (Year) | Q1^a^ | Q2 | Q3 | Q4 | Q5 | Q6 | Q7 | Q8 | % Yes | Risk^b^ |
| --- | --- | --- | --- | --- | --- | --- | --- | --- | --- | --- |
| Davis 2022 | Y | Y | Y | Y | Y | Y | Y | Y | 100% | Low |
| Gospodarevskaya 2013 | Y | Y | Y | Y | Y | Y | Y | Y | 100% | Low |
| Ferry 2015 | Y | Y | Y | Y | Y | Y | Y | Y | 100% | Low |
| Noh 2019 | Y | Y | Y | Y | Y | Y | Y | Y | 100% | Low |
| Le 2018 | Y | Y | Y | Y | Y | Y | Y | Y | 100% | Low |
| Roggenkamp 2021 | Y | Y | Y | Y | Y | Y | Y | Y | 100% | Low |
| Lorenzoni 2024 | Y | Y | Y | Y | Y | Y | Y | Y | 100% | Low |

^a^Q1 – Q8 indicate questions 1 to 8 based on the JBI critical appraisal checklist for analytical cross sectional studies.

^b^The risk of bias was ranked as high when the study reached up to 49% of “yes” scores, moderate when the study reached from 50 to 69% of “yes” scores, and low when the study reached more than 70% of “yes” scores. ‘Y’ indicates yes, ‘N’ indicates no, ‘U’ indicates unclear and ‘N/A’ indicates unclear not applicable.
