# Supplementary material for: Economic and health-related quality of life impacts of posttraumatic stress disorder: a systematic review
Source: Health Econ Rev. 2026 Jun 2;16:93. doi: 10.1186/s13561-026-00800-5 (PMC13463949; doi:10.1186/s13561-026-00800-5)
Supplement: Supplementary file 3 [file 13561_2026_800_MOESM3_ESM.docx]

**Economic and health-related quality of life impacts of posttraumatic stress disorder: A systematic review**

# Search strategy

**PubMed**

Search: ("stress disorders, post traumatic"[MeSH Terms] OR "stress disorder*"[Title/Abstract] OR "acute stress disorder"[Title/Abstract] OR "PTSD"[Title/Abstract] OR "Disorders of extreme stress not otherwise specified"[Title/Abstract] OR "DESNOS"[Title/Abstract]) AND ("Cost of illness"[Title/Abstract] OR "health expenditure*"[Title/Abstract] OR "out of pocket payment*"[Title/Abstract] OR "OOP"[Title/Abstract] OR "expenditure*"[Title/Abstract] OR "cost"[Title/Abstract] OR "spending"[Title/Abstract] OR "expense*"[Title/Abstract] OR "health cost*"[Title/Abstract] OR "direct cost*"[Title/Abstract] OR "indirect cost*"[Title/Abstract] OR "economic"[Title/Abstract] OR "cost effectiveness"[Title/Abstract] OR "Economic evaluation"[Title/Abstract] OR "willingness to pay"[Title/Abstract] OR "productivity loss*"[Title/Abstract] OR "Cost benefit analysis"[Title/Abstract] OR "Cost utility analysis"[Title/Abstract] OR "Cost of illness"[Title/Abstract] OR "economic outcome*"[Title/Abstract] OR QALY[MeSH Terms] OR "Quality adjusted life year*" OR DALY OR "Disability adjusted life year*") - Saved search Sort by: Most Recent

**MEDLINE, EMBASE, and PsycINFO (via Ovid)**

1.	Stress Disorders, Post-Traumatic/

2.	"stress disorder*".mp.

3.	"acute stress disorder".mp.

4.	PTSD.mp.

5.	"Disorders of extreme stress not otherwise specified".mp.

6.	DESNOS.mp.

7.	1 or 2 or 3 or 4 or 5 or 6

8.	"cost of illness".mp.

9.	"Health expenditure*".mp.

10.	"out‐of‐pocket payment".mp.

11.	OOP.mp.

12.	expenditure.mp.

13.	cost.mp.

14.	spending.mp.

15.	expense.mp.

16.	"health cost*".mp.

17.	"direct cost*".mp.

18.	"indirect cost*".mp.

19.	economic.mp.

20.	"cost effectiveness".mp.

21.	"Economic evaluation".mp.

22.	"willingness to pay".mp.

23.	"Productivity loss*".mp.

24.	"Cost –benefit analysis".mp.

25.	"Cost utility analysis".mp.

26.	"Cost of illness".mp.

27.	"economic outcome*".mp.

28.	"cost effectiveness".mp.

29.	QALY.mp. or Quality-Adjusted Life Years/

30.	DALY*.mp.

31.	disability adjusted life year*.mp.

32.	8 or 9 or 10 or 11 or 12 or 13 or 14 or 15 or 16 or 17 or 18 or 19 or 20 or 21 or 22 or 23 or 24 or 25 or 26 or 27 or 28 or 29 or 30 or 31

33.	7 and 32

**Web of Science**

1.	(((((TS=(stress disorders, post traumatic)) OR TS=(stress disorder*)) OR TS=("acute stress disorder")) OR TS=("PTSD")) OR TS=("Disorders of extreme stress not otherwise specified")) OR TS=("DESNOS")

2.	((((((((((((((((((((((TS=("Cost of illness")) OR TS=("health expenditure*")) OR TS=("out of pocket payment*")) OR TS=("OOP")) OR TS=("expenditure*")) OR TS=("cost")) OR TS=("spending")) OR TS=("expense*")) OR TS=("health cost*")) OR TS=("direct cost*")) OR TS=("indirect cost*")) OR TS=("economic")) OR TS=("cost effectiveness")) OR TS=("Economic evaluation")) OR TS=("willingness to pay")) OR TS=("productivity loss*")) OR TS=("Cost benefit analysis")) OR TS=("Cost utility analysis")) OR TS=("Cost of illness")) OR TS=("economic outcome*")) OR TS=("Quality adjusted life year*")) OR TS=("DALY")) OR TS=("Disability adjusted life year*")

3.	#1 AND #2

**EconLit and NHS Economic Evaluation Database**

Same with PubMed

# Inclusion/ exclusion criteria

## Participants/Population

**Inclusion**

- Any individuals who was/is suffering from PTSD* regardless of treatment status (treated/untreated) and source of trauma.
  - Subgroups: source of trauma (cause); age group; sex; ethnicity; socioeconomic status; geographical location (e.g., country); occupation/natural of job (e.g., civilian/military personnel/healthcare professional).

* An individual is considered to have PTSD if it was diagnosed and marked by ICD-9 (309.81) or ICD-10 (F43.1) or equivalent in the patient’s medical record

**Exclusion**

- None

## Interventions

**Inclusion**

- Any treatment for PTSD
  - Recommended psychological therapies (by NICE): Trauma-focused cognitive behavioural therapy (TF-CBT) and Eye movement desensitisation and reprocessing (EMDR).
  - Medications (e.g., antidepressant, antipsychotic etc.)
  - Group therapy, arts therapies, or dialectical behaviour therapy (DBT).

**Exclusion**

- None

## Comparators

- Any comparators (e.g., individuals without PTSD, individuals with PTSD but received different intervention)

## Study design

- Inclusion: Any study designs except those listed below
- Exclusion: Systematic reviews, letter, commentary, modelling studies without real world data, conference proceedings (with no full text), study with only abstract, study protocol.

## Context

Any countries will be included.

Subgroups: High income countries (HICs) and Low- and middle-income countries (LMICs); countries with and without on-going war/conflict.

## Main outcome(s)

- Cost (including direct medical costs, direct non-medical costs, and indirect costs)
- Health-related quality of life in the forms of QALYs and/or DALYs (Studies reported any other outcomes of HRQoL will be excluded)

## Additional outcome(s)

Other relevant outcomes such as mortality, self-harm, mental illness measure etc.
